# Diagnosis of Obstructive Sleep Apnea in Parkinson's Disease Patients: Is Unattended Portable Monitoring a Suitable Tool?

**DOI:** 10.1155/2015/258418

**Published:** 2015-10-13

**Authors:** Priti Gros, Victoria P. Mery, Anne-Louise Lafontaine, Ann Robinson, Andrea Benedetti, R. John Kimoff, Marta Kaminska

**Affiliations:** ^1^Respiratory Division and Sleep Laboratory, McGill University Health Centre, Montreal, QC, Canada H4A 3J1; ^2^Clinica Alemana de Santiago, Facultad de Medicina Clinica Alemana, Universidad del Desarrollo, Santiago, Chile; ^3^Montreal Neurological Hospital, McGill University Centre, Montreal, QC, Canada H3A 2B4; ^4^Department of Medicine and Department of Epidemiology, Biostatistics & Occupational Health, McGill University Health Centre, Montreal, QC, Canada H4A 3J1; ^5^Respiratory & Epidemiology and Clinical Research Unit, McGill University Health Centre, Montreal, QC, Canada H4A 3J1

## Abstract

*Purpose.* Obstructive sleep apnea (OSA) is frequent in Parkinson's disease (PD) and may contribute to nonmotor symptoms. Polysomnography (PSG) is the gold standard for OSA diagnosis. Unattended portable monitoring (PM) may improve access to diagnosis but has not been studied in PD. We assessed feasibility and diagnostic accuracy in PD. *Methods.* Selected PD patients without known OSA underwent home PM and laboratory PSG. The quality of PM signals (*n* = 28) was compared with matched controls. PM accuracy was calculated compared with PSG for standard apnea hypopnea index (AHI) thresholds. *Results.* Technical failure rate was 27.0% and airflow signal quality was lower than in controls. Sensitivity of PM was 84.0%, 36.4%, and 50.0% for AHI cut-offs of 5/h, 15/h, and 30/h, respectively, using the same cut-offs on PM. Specificity was 66.7%, 83.3%, and 100%, respectively. PM underestimated the AHI with a mean bias of 12.4/h. Discrepancy between PM and PSG was greater in those with more motor dysfunction. *Conclusion.* PM was adequate to “rule in” moderate or severe OSA in PD patients, but the failure rate was relatively high and signal quality poorer than in controls. PM overall underestimated the severity of OSA in PD patients, especially those with greater motor dysfunction.

## 1. Introduction

In Parkinson's disease (PD), sleep-related problems are one of the most prevalent nonmotor symptoms (NMS), affecting 48 to 82% of patients and increasing with the disease severity [1]. Among them, obstructive sleep apnea (OSA) is common and is thought to occur in 20 to 60% of PD patients [2–5]. OSA is characterized by recurrent complete (apnea) or partial (hypopnea) upper airway obstruction resulting in intermittent hypoxemia and arousals from sleep. It is known to cause neurocognitive dysfunction, cardiovascular complications, and metabolic disorders in the general population [6]. Recent preliminary data suggest that, in PD patients, OSA appears to worsen other NMS, such as cognitive dysfunction and excessive daytime sleepiness [7]. Treatment of OSA could be a strategy to help improve important NMS, such as excessive daytime sleepiness [2]. Hence, early diagnosis and therapy for OSA could result in better outcomes for PD patients.

Polysomnography (PSG), or level 1 sleep testing, is currently the gold standard for OSA diagnosis [8, 9]. It includes at least 7 channels of data (typically ≥16) and requires an overnight stay in the sleep laboratory. It allows assessment of sleep-wake stages (EEG, EOG, and EMG), nasal airflow, snoring, respiratory efforts, oxygen saturation, body position and movements, cardiac electrical signals (EKG), and others when necessary. PSG is complex, expensive, and poorly accessible. In Canada, the waiting time to access PSG studies varies from 8 to 36 months [10]. In the United States, it varies from 2 to 10 months [10]. There is growing interest in novel diagnostic tools and methodologies, such as American Academy of Sleep Medicine (AASM) level III testing which uses portable monitoring (PM) conducted in an unattended setting. A variety of different devices recording different signals are available [11]. The AASM recommends unattended PM use as a diagnostic tool for patients with a high pretest probability of moderate to severe OSA, with no major comorbidities and/or other sleep disorders [9]. In this context, a meta-analysis of 19 studies by Shayeb et al. found that sensitivity and specificity were generally both good, with increasing specificity and decreasing sensitivity as the disease severity increased [11]. The sensitivities and specificities were, respectively, 93% and 60% for AHI ≥ 5/h, 79% and 79% for AHI ≥ 15/h, and 79% and 90% for AHI ≥ 30/h [11]. Cost-effectiveness studies have suggested a decreased cost for PM of up to one-half compared to PSG [12].

In 2008, the US Centers for Medicare and Medicaid Services (CMS) committee released a landmark decision regarding the National Coverage Determination (NCD), approving “Home Sleep Testing (HST)” as a means to qualify patients with OSA for continuous positive airway pressure (CPAP) therapy [13]. This opens the way for more widespread use of PM. PM is an attractive alternative in patients with neurological disorders such as PD, who might otherwise decline in-laboratory PSG due to difficulties related to their disease such as impaired mobility, bladder dysfunction, anxiety, and cognitive impairment. However, PM is performed in an unattended setting, which can increase the rate of technically suboptimal studies, particularly in patients with motor or cognitive impairment. Furthermore, reduced sleep efficiency (i.e., greater proportion of wakefulness during recording) can lead to underestimation of the AHI on PM in that event indices are calculated based on recording rather than sleep time as no EEG is recorded [14]. Similarly, EEG arousals that are needed for scoring of some hypopneas cannot be detected on PM resulting in potential underestimation of OSA severity. Scoring so-called autonomic arousals (pulse accelerations) as a surrogate for EEG arousal can help improve sensitivity of PM for detection of OSA [15].

The feasibility and accuracy of PM have not been assessed in patients with PD. The objective of this prospective cohort study was therefore to assess the feasibility (quality of signals, study failure rates) and diagnostic accuracy of PM performed at home, compared with the gold standard of in-laboratory PSG, in PD patients with suspected OSA.

## 2. Materials and Methods

### 2.1. Study Subjects

PD patients with sleep complaints were recruited between November 2011 and July 2014 from the McGill Movement Disorder Clinic, an academic tertiary care centre. Inclusion criteria were a clear diagnosis of primary PD (as per established criteria [16, 17]), ability to undergo polysomnography (PSG), and adequate knowledge of English or French. Patients were excluded if they had another major neurological disorder (e.g., stroke), unstable cardiac disease, uncontrolled hypertension, an expected survival of <6 months, psychiatric or cognitive impairment precluding informed consent, or previously diagnosed OSA. Patients with other sleep disorders such as rapid eye movement sleep behavior disorder (RBD) or restless leg syndrome (RLS) were not excluded. Patients remained on their usual PD treatment regimen during the study. PM was offered to a selected group of patients based on their availabilities and their subjective capacity to use the device. Control PM studies performed on patients without major medical comorbidities referred to our general sleep-disorders clinic for suspected OSA were identified from our clinical sleep laboratory records. Records from the same period of time were reviewed sequentially until 2 control studies for each PD subject were identified, based on the same sex and age ±3 years.

### 2.2. Study Design

A prospective study protocol was used, in which patients completed both a PSG night and a PM night, separated by less than 30 days. The order in which these were done depended on subject availability. Prior to the PM night, patients were instructed by the research assistant on the correct use of the device, which was programmed to start recording automatically at the subjects' usual bedtime. The study visit consisted of a baseline questionnaire and a brief physical exam. Spirometry was performed according to American Thoracic Society guidelines; forced vital capacity (FVC) and forced expiratory volume in one second (FEV1) were measured [18].

Factors possibly affecting PM performance in PD were assessed. The Movement Disorder Society-sponsored revision of the Unified Parkinson Disease Rating Scale (MDS-UPDRS) was used to assess motor dysfunction [19]. A higher score is associated with a more severe PD. Cognitive impairment was assessed with the Montreal Cognitive Assessment [20]. A score of <26 is generally considered suggestive of cognitive impairment. We assessed dysautonomia using question 1.12 from the MDS-UPDRS, which evaluates light-headedness on standing (scores 0–4). A score of 0 represents no dysautonomia, whereas a score of 1 to 4 represents dysautonomia.

### 2.3. Measurements

#### 2.3.1. Polysomnography

Patients underwent standard overnight polysomnography, using a 6-channel recording system (C3, C4, F3, F4, O1, and O2), bilateral tibialis anterior and extensor digitorum electromyography (EMG), and digital video. Respiratory inductance plethysmography was used for thoracoabdominal motion, and nasal pressure cannula measured airflow. Oxygen saturation (SpO_2_) was continuously monitored with a finger oximeter. Total sleep duration of minimum 3 hours during PSG was required. Data for PSG was scored manually by one certified registered polysomnographic technician using standard American Academy of Sleep Medicine (AASM) clinical criteria [21] for all measures except respiratory events, which were scored using AASM research criteria (Chicago criteria) [22]. The software Stellate Harmony (Natus, Mississauga, Canada) was used. The scoring was subsequently reviewed by an expert sleep physician. Outcomes of interest were apnea hypopnea index (AHI), respiratory arousal index (RAI), periodic limb movement arousal index (PLMAI), total arousal index (TAI), and oxygen desaturation index (≥4%, ODI_PSG_).

#### 2.3.2. Level III Home Portable Monitoring

Type III home portable monitoring (Embletta Gold Natus Medical Incorporated, San Carlos, CA, USA) was used. It included two respiratory inductance plethysmography belts, a nasal pressure cannula and a pulse oximeter. The machine was preset by the research assistant. Data from the PM was scored manually by the same certified technician who was blinded to the PSG results and subsequently reviewed by an expert sleep physician. Embla RemLogic software was used. Scoring was based on the “Chicago criteria” [22] used in our laboratory for PSG, modified for PM recordings. Apnea was defined as a cessation (≥90% decrease from baseline) of nasal airflow for at least 10 seconds. Hypopnea was defined as a clear decrease of nasal airflow from baseline (but <90%) lasting at least 10 seconds accompanied by either an oxygen desaturation ≥4% or a transient pulse acceleration ≥6 beats/min (bpm) as a surrogate marker for EEG arousal [15] (“autonomic hypopnea”), or a decrease in flow ≥50% with neither desaturation nor pulse increase. The respiratory disturbance index (RDI) was calculated as the number of apneas and hypopneas per hour of recording. An oxygen desaturation index (ODI_PM_) was also calculated.

### 2.4. Data Analysis

Baseline demographic and polysomnographic data were described with means and standard deviations (SD). The Shapiro-Wilk test was used to test the normality of our data. Simple univariable comparisons between groups were performed with Student's *t*-test when the data were normally distributed, or the Mann-Whitney *U* test (MWU) if they were not. *χ* ^2^ or the Fisher exact tests were used as appropriate to compare nominal scale variables. Linear regressions adjusted for age and gender were performed as well.

The primary outcome of interest was the feasibility of PM studies in PD patients. The proportion of failures was estimated as well as its 95% confidence intervals (95% CI). A study was considered a failure when no signal at all was available on the recording for all channels. Quality of the PM recordings was assessed with the total recording time (minutes), the airflow signal quality (% of optimal signal), the oxygen saturation signal quality (% of optimal signal), and the pulse signal quality (% of optimal signal) as provided by the RemLogic software and was compared between cases and controls. We assessed correlations between signal quality and age as well as PD parameters: Hoehn and Yahr score, PD duration, motor part of the UPDRS, Montreal Cognitive Assessment (MOCA) score, and dysautonomia score, using the Pearson correlation coefficient. The secondary outcome of interest was the diagnostic performance of the PM device to rule in or rule out OSA in PD patients. Standard cut-offs for AHI as measured on PSG (gold standard) were evaluated: AHI ≥ 5/h (mild OSA), AHI ≥ 15/h (moderate OSA), AHI ≥ 30/h (severe OSA), and ODI ≥ 5/h. Receiver operator characteristic (ROC) curves and a Bland-Altman plot were built. To evaluate whether specific patient characteristics affected the accuracy of PM recordings, we assessed agreement between RDI and AHI, using the RDI/AHI ratio, comparing by *t*-test those with and without specific characteristics including age (dichotomized at the median), motor dysfunction (MDS-UPDRS dichotomized at the median), cognitive dysfunction (MOCA < 26 versus ≥26), the presence of dysautonomia, and negative chronotropic medications. Data were analyzed using SPSS statistics version 22.0 and SAS version 9.3 (SAS Institute, Inc., Cary, North Carolina, 2010). Statistical significance was defined at the 5% level.

## 3. Results

### 3.1. Subjects' Characteristics

**Figure 1:**
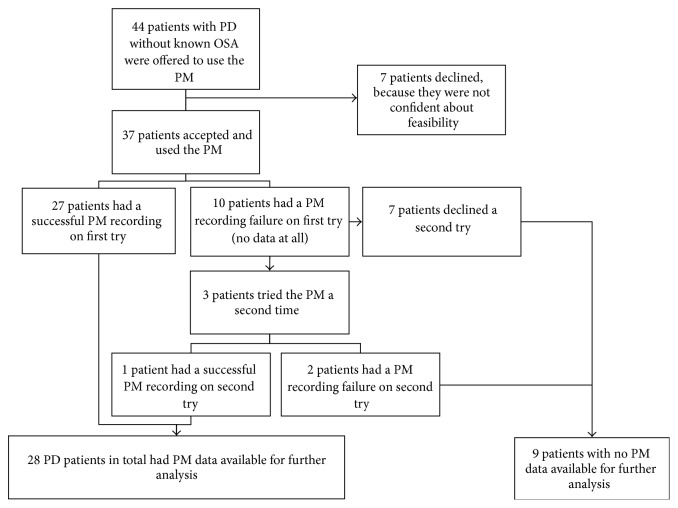
Patients' recruitment flow diagram.

Of the 44 PD patients recruited, 7 declined because they were not confident about their ability to install the PM. From the 37 patients who used the device, 10 had a recording failure with no signal at all. Of those, 3 patients accepted a second attempt and one patient had a subsequent successful recording (Figure 1).

Patient characteristics are shown in Table 1. PD patients with a successful PM recording had a Parkinson's disease duration of 5.3 years (±5.2) on average, with a Hoehn and Yahr stage range from 1.0 to 4.0. The average Montreal Cognitive Assessment (MOCA) score of 25.4 (±3.7) and 39.3% had a MOCA score < 26, suggestive of cognitive impairment. None had frank dementia. Patients were on their usual PD medication during the study.

Subjects had OSA of moderate severity on average on PSG (AHI 28.2 ± 19.5/h). The obstructive apnea index was 2.5 ± 4.6 events/h; the central apnea index was 1.1 ± 2.8 events/h (Table 2). Most of the respiratory events were hypopneas with arousal, but there was little associated hypoxemia; the respiratory arousal index (RAI) was 24.3 ± 15.8/h and the oxygen desaturation index (≥4%, ODI_PSG_) was only 7.3 ± 12.4/h. From PM recordings, the mean RDI was 15.0/h and the ODI_PM_ was 6.5/h (Table 3). “Autonomic hypopneas” represented 31.6% of the RDI.

### 3.2. Feasibility of PM in PD Patients

**Figure 2:**
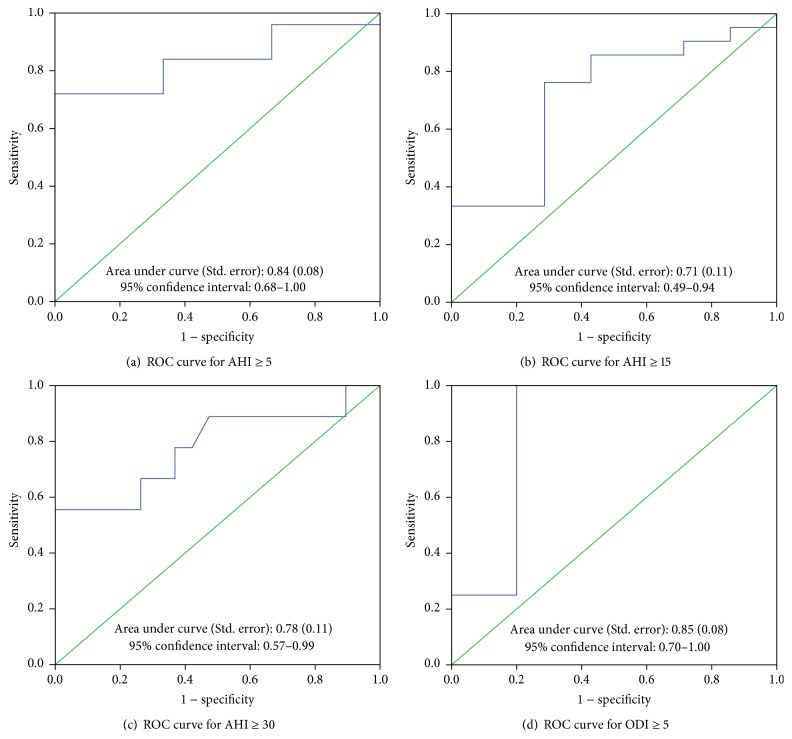
Receiver operating characteristics curve analysis of PM for AHI ≥ 5, AHI ≥ 15, AHI ≥ 30, and ODI ≥ 5. ROC referred to an AHI cut-off from PSG, which shows the sensitivity and specificity of each observed value of the RDI obtained from the PM in relation to the given PSG cut-off.

Feasibility of PM was assessed with the technical failure rate and the PM signal quality. Technical failure occurred in 27.0% of patients on their first attempt (Figure 1) and 2 of 3 (67%) on the second attempt. There were no significant differences in the demographic characteristics and in the polysomnographic data between the PD patients with a successful PM recording and those with a recording failure, except for the BMI, which was lower, and the percentage of sleep time in supine, which was higher for subjects with recording failure (Tables 1 and 2). There was a trend for lower PM signal quality in PD patients compared to controls. However, airflow signal quality recording was significantly lower in PD patients.

Correlation coefficients were calculated between the quality of the signals (proportion of adequate signal) and age (Table 4) as well as PD-specific variables (Table 7, in Supplementary Materials available online at http://dx.doi.org/10.1155/2015/258418). Trends were observed for negative correlations between age and quality of signals in PD patients. However, there was no significant correlation between quality of signals and the Hoehn and Yahr score, PD duration, MDS-UPDRS motor score, MOCA score, or dysautonomia.

### 3.3. Performance of PM in PD Patients

The diagnostic performance of the PM in categorizing mild, moderate, and severe OSA is presented in Table 5. The sensitivity of the PM was generally poor, except for AHI ≥ 5/h with a sensitivity of 84% (95% CI: 64%–95%). The specificity of PM was relatively high and reached 100% (95% CI: 82%–100%) for AHI ≥ 30/h. The positive predictive value was consistently high for all AHI cut-offs in our population. The negative predictive value (NPV) was poor except for AHI ≥ 30/h where it was 83% (95% CI: 61%–95%). The accuracy was above 80% for AHI ≥ 5/h and for AHI ≥ 30/h in our subject group.

For AHI ≥ 15/h, considered the most clinically relevant cut-off, several RDI cut-off values were evaluated to try to improve diagnostic accuracy: RDI ≥ 10/h, RDI ≥ 15/h, and RDI ≥ 20/h (Table 5). Sensitivity was doubled when RDI ≥ 10/h was used compared to the RDI ≥ 15/h cut-off, as there were less false negatives, with only mildly reduced specificity. On the other hand, specificity reached 100% when RDI ≥ 20/h was used compared to RDI ≥ 15/h, as there were less false positives, but sensitivity was poor.

The performance of ODI_PM_ ≥ 5/h as compared to ODI_PSG_ ≥ 5/h gave a different pattern than for AHI. It was more sensitive than specific and had a higher NPV than PPV. The overall accuracy was 78.6%. Receiver operating characteristics (ROC) curves for the different AHI cut-offs are shown in Figure 2. The best area under the curve (0.84; 95% CI: 0.68–1.00) corresponded to the AHI ≥ 5/h cut-off. The area under curve for ODI_PM_ ≥ 5/h was also high (0.85; 95% CI = 0.70–1.00).

### 3.4. Agreement between PM Studies and PSG

**Figure 3:**
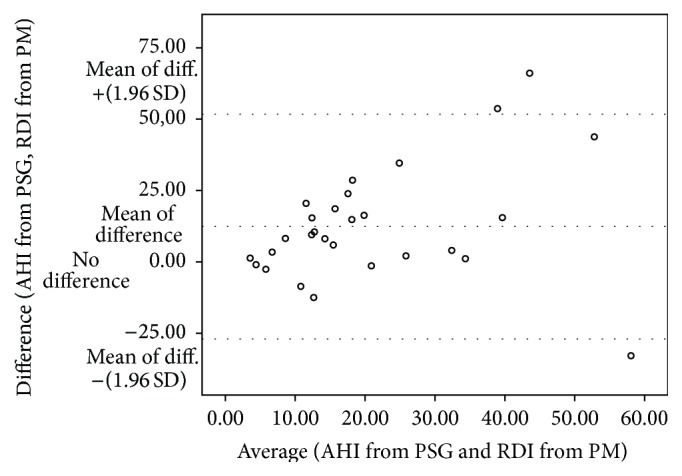
Bland-Altman plot comparing AHI from PSG and RDI from PM.

The Bland-Altman plot provides a visual representation of the agreement between PSG and PM (Figure 3). The mean difference (AHI from PSG, RDI from PM) was positive at 12.4 ± 20.1/h. In most cases, the PM underestimated the AHI, with only 14% of the data points below the line of no difference, suggesting a minority of overestimations by the PM. The difference between the two measures increased with increasing severity of OSA.

### 3.5. Effect of Patient Characteristics on Performance

We compared the mean RDI/AHI ratio in patients with and without certain characteristics that could affect PM performance, including older age, higher MOCA score, higher MDS-UPDRS motor score, dysautonomia, and negative chronotropic medication (Table 6). The two latter factors were chosen as they were thought to potentially affect the detection of autonomic arousals used in the scoring of hypopneas (cf. Materials and Methods). Increased motor dysfunction was associated with a significantly lower RDI/AHI ratio. Presence of dysautonomia and negative chronotropic medication was also associated with lower RDI/AHI ratio (not statistically significant).

## 4. Discussion

We found that PM was feasible in a selected PD population, although the rate of complete technical failures was relatively high, and airflow signal quality was lower than in the control group. The PM had generally low sensitivity but high specificity for various cut-offs of AHI, making it an adequate tool to “rule in” but not “rule out” OSA in PD patients. Overall, the RDI was an underestimate of the AHI. The PM had an excellent sensitivity for ODI ≥ 5/h.

The failure rate among subjects who attempted an initial PM study was 27.0% in our group of PD patients. This is higher than the rate of 10.3% previously reported in the general sleep clinic population [11] and than in our clinical laboratory, 1% to 7% per month for the same time period [23]. Moreover, 7 of 44 (16%) PD patients who were offered PM testing declined and only 30% of those who had a technical failure agreed to repeat the study. Most patients declined because they felt self-installation of the PM was too overwhelming. Psychiatric symptoms associated with PD such as anxiety or depression could be another factor related to the high noncompletion rate. Of note, these patients also underwent PSG as part of the study protocol. In the clinical setting, patients might theoretically prefer to undergo or repeat a PM study rather than undergoing PSG.

Although the quality of the airflow and pulse oximetry signals was overall adequate, there was a significantly lower airflow signal quality for PD patients compared to controls. It is possible that motor symptoms or cognitive dysfunction present in PD could impede the proper installation of the device or more readily lead to displacement of the nasal cannula during the night. However, neither the PD motor severity variables nor the MOCA score correlated with signal quality (Table 7, supplementary materials). Age appears to play a role in PD patients but not in control subjects (Table 4) with respect to signal quality. However, this does not appear to affect performance of the PM as RDI/AHI ratio was no different in younger versus older patients (Table 6). We did not systematically assess whether the patients installed the PM device themselves or if a caregiver helped them, but this might have affected willingness to undergo PM testing as well as signal quality.

Our data suggest that PM is a good tool to rule in OSA in PD patients with a prior clinical suspicion, as the specificity and the PPV were high. Specificities for PM in PD patients were roughly equivalent to those reported in the general population [11]. Patients with a PM recording suggestive of moderate or severe OSA are most likely to have OSA. This is consistent with the current AASM recommendation that PM may be used to rule in OSA in patients with a high pretest probability of moderate to severe OSA [9]. However, it does not seem to be an adequate tool to rule out OSA. The sensitivities and NPVs of the PM were poorer for PD patients. There was a high rate of false negatives, higher than the 4 to 8% of false negative reported by the AASM for unattended type III PM [9]. The PM tends to underestimate severity of OSA, as seen on the Bland-Altman plot. The mean bias (AHI-RDI) was 12.4 ± 20.1/h. Discrepancy between RDI and AHI was significantly greater in patients with more marked motor dysfunction (Table 6). Scoring of respiratory events on PM recordings in these patients may be more challenging in the absence of EEG.

An important factor that could contribute to the increased PM false negative rate is the type of OSA found in PD patients. In our population, most events on PSG were hypopneas with arousals but few desaturations. This is likely in part due to lower BMI of our study subjects compared to general population with OSA, which has been shown to result in less desaturation in association with apnea and hypopnea during sleep [24]. However, on PM, EEG is not recorded and arousals are not scored. Cascon et al. found that heart rate increases associated with autonomic hypopneas considered as a surrogate marker of cortical arousals could improve the diagnostic accuracy of OSA with PM [15]. This has been standard in our sleep laboratory for some years. However, PD patients frequently have dysautonomia, which could undermine the accuracy of the PM by blunting heart rate responses. In our cohort, 43% had dysautonomic features, with self-reported light-headedness. Although this is a subjective and imprecise assessment of dysautonomia, those patients appeared to have greater discrepancy between RDI and AHI. Moreover, Lachapelle et al. have suggested that negative chronotropes could interfere significantly with “autonomic hypopnea” detection and consequently with PM accuracy [25]. Of our patients, 18% were on either beta-blockers or calcium channel blockers. Although we did not have sufficient power to demonstrate a statistically significant effect, our data suggest that these two factors could affect the accuracy of PM recordings by impeding measurement of pulse accelerations needed to score some hypopneas causing underestimation of events. It is also important to note that laboratories that do not score “autonomic hypopneas” and rely solely on oxygen desaturation to score hypopnea events on PM would most likely significantly underestimate the AHI, resulting in lower sensitivity for OSA diagnosis.

None of the patients in our study had any hypoventilation or predominant central sleep apnea. This is similar to the findings of Cochen De Cock et al. [3]. However, others [26] have found a number of PD patients with predominant central sleep apnea. This may depend on the patient population, comorbidities, and PD medications [26].

Overestimation of AHI occurred in 14% of the PM studies. This could be due to artefacts that would be more difficult to appreciate on PM recordings than on PSG. Sleep staging is not performed with PM, and respiratory fluctuations in wakefulness may have been scored as sleep-disordered breathing. Moreover, the PM and PSG studies were run on separate nights, so the night-to-night variability in OSA severity may have contributed to discrepancies [27].

*Strengths and Limitations*. We studied the PM in its site of intended use, the patient's home, which aimed at representing performance in real practice. PSG and PM studies were scored by the same sleep technician, in a blind manner, helping to prevent interscorer variability and observational bias. Our experimental design simulated the clinical use of PM in our sleep laboratory, with instructions regarding the installation of the PM identical to the standard clinical setting. This makes our results generalizable to the average sleep laboratory setting.

There were some limitations to our study. PSG and PM in the PD patients were done on two different nights, in two different environments. Although these are more “real-life” conditions, differences in results may be explained at least in part by night-to-night variability which is known to occur in OSA in the general population [27]. However, a previous study suggested OSA in PD appears to be relatively stable across different nights [28]. Further research with simultaneous PM and PSG on the same night could be relevant to better assess this factor. In this study, we have not excluded patients with RLS. We had a relatively high number of positive questionnaires for RLS, but we did not have clinical confirmation of RLS diagnosis. The questionnaire may overestimate RLS due to inclusion of RLS mimics by the questionnaire. We found that the periodic limb movements of sleep index (PLMS) and the PLM-related arousal index (PLMAI) were relatively low (Table 2). Therefore, we expect PLM to only have a minor influence on our data. The study sample was relatively small and our population may not be entirely representative of all PD patients, which can affect external validity. We excluded patients that could not undergo in-laboratory PSG, thereby excluding most advanced PD patients. Patients could also decline to undergo PM if they did not feel capable of doing it. Hence, our results apply to a selected population of PD patients. This study was observational and not experimental and there remains the potential for unknown and unmeasured bias.

*Clinical Relevance*. Our results can be applied to a clinical population of PD patients with sleep complaints. When a PM study is positive for moderate or severe OSA in a PD patient, it is likely that the patient has clinically significant OSA and could benefit from treatment, such as CPAP. Treating OSA in PD could help improve NMS, such as excessive daytime sleepiness [2, 29]. Conversely, when a PM study is negative, mild and moderate OSA have not been excluded, although severe OSA is less likely. A full PSG should then be considered in this case. PSG should also be performed when other sleep disorders are suspected.

## 5. Conclusion

To our knowledge, this is the first study attempting to validate the use of PM in PD patients and as such it addresses a knowledge gap in the literature. There is recently a trend for increased use of PM for OSA diagnosis. It is important to understand implications of using this type of sleep testing in PD. Additionally, PM use may help simplify access to OSA diagnosis in PD, since PSG has limited availability and may represent a significant burden in PD patients. Our study suggests that PM is feasible in some PD patients, although the failure rate was higher and the signal quality was lower than in a general sleep clinic population. Increasing age was associated with poorer signal quality but this did not affect the agreement between RDI and AHI. However, in patients with greater motor dysfunction and in those with dysautonomia or on negative chronotropic medications, the severity of OSA was underestimated by the PM (lower RDI/AHI ratio). Overall, PM is a good tool to rule in OSA in PD patients with moderate or severe OSA. In the context of increasing evidence implicating OSA as a potentially harmful and frequent comorbidity in PD, increasing the use of home testing in clinical practice when appropriate could facilitate a prompt diagnosis and treatment of OSA. The limitations of PM performance have to be taken into account when deciding on its use and when interpreting PM recordings, including its inability to detect sleep disorders other than OSA. Additional studies with larger cohort are needed to confirm these findings and to assess the cost-effectiveness of strategies employing PM testing among PD patients.

## Supplementary Material

Table 7 in Supplementary Material describes the correlation between the portable monitoring signals quality with several Parkinson's disease associated variables.

## Figures and Tables

**Table 1:** Patient baseline characteristics.

	PD patients with successful PM recording (*n* = 28)	PD patients with PM recording failure (*n* = 9)	PD patients who declined PM (*n* = 7)	Controls (*n* = 56)
*Clinical data*				
Sex (% male)	71.4	55.6	57.1	71.4
Age (years)	64.6 (11.0)	65.8 (11.2)	66.4 (8.2)	64.9 (10.4)
Body Mass Index (kg/m^2^)	27.9 (3.7)	24.1 (3.2)	28.3 (4.0)	—
Hoehn and Yahr	2.0 (0.9) *(Range: 1.0 to 4.0)*	1.9 (1.0) *(Range: 1.0 to 4.0)*	2.4 (0.4) *(Range: 2.0 to 3.0)*	—
Total UPDRS	52.5 (25.2)	46.2 (24.0)	45.7 (14.3)	—
Motor UPDRS	25.0 (14.1)	19.3 (13.2)	20.6 (8.7)	—
PD duration (years)	5.3 (5.2)	5.6 (2.7)	6.9 (5.9)	—
MOCA score	25.4 (3.7) *(Range: 18 to 30)*	24.9 (3.1) *(Range: 20 to 29)*	25.0 (2.9) *(Range: 22 to 30)*	—
Levodopa equivalence dose (mg/day)	701.2 (902.3)	804.9 (522.1)	609.1 (210.0)	—
Proportion (%) on negative chronotropic medication°	17.8	22.2	14.3	—
Proportion (%) with dysautonomia^¶^	42.9	66.7	42.9	—
FVC (L)	4.0 (1.0)	3.6 (1.0)	3.2 (0.9)	—
FVC % pred.	109 (26.2)	102.6 (11.6)	99.3 (10.5)	—
FEV1 (L)	3.0 (0.8)	2.7 (0.7)	2.3 (0.7)	—
FEV1 % pred.	103 (25.7)	95.9 (11.3)	87.0 (17.0)	—

°Negative chronotropes include either beta-blockers or calcium channels blockers.

^¶^The dysautonomia score is based on a question from the first part of the UPDRS (see Section 2), regarding light-headedness on standing. Patients with reported dysautonomia have slight to severe symptoms, whereas patients with no reported dysautonomia have no symptoms of light-headedness.

PM: portable monitoring.

PD = Parkinson's disease.

UPDRS: Unified Parkinson's Disease Rating Scale.

PD duration: number of years since PD diagnosis.

MOCA: Montreal Cognitive Assessment.

FVC: forced vital capacity.

FEV1: forced expiratory volume in one second.

**Table 2:** Polysomnographic data.

	PD patients with successful PM recording (*n* = 28)	PD patients with PM recording failure (*n* = 9)
Polysomnographic data		
Total sleep time (min)	333.3 (59.9)	334.3 (56.9)
Sleep efficiency (%)	76.3 (12.5)	78.5 (14.0)
Wake after sleep onset (min)	89.5 (57.2)	90.5 (67.1)
Stage changes	177.9 (65.9)	161.6 (40.3)
Stage 1 (% TST)	13.8 (11.6)	10.0 (5.2)
Stage 2 (% TST)	50.9 (14.5)	51.4 (15.3)
Stage 3 (% TST)	23.3 (16.4)	26.0 (17.8)
Stage REM (% TST)	12.0 (8.1)	12.7 (9.4)
% Total sleep time in supine position	59.9 (29.5)	47.1 (18.7)
Total arousal index (events/h)	43.0 (17.7)	37.6 (13.6)
Respiratory arousal index (events/h)	24.3 (15.8)	16.9 (11.3)
Periodic limb movements of sleep index (events/h)	19.6 (21.5)	59.1 (65.3)
Periodic limb movements arousal index (events/h)	2.8 (3.5)	5.6 (5.8)
Spontaneous arousal index (events/h)	15.5 (6.2)	15.0 (4.6)
AHI (events/h)	28.2 (19.5)	20.4 (13.0)
Proportion (%) with AHI ≥ 5	89.3	87.5
Proportion (%) with AHI ≥ 15	78.6	62.5
Proportion (%) with AHI ≥ 30	35.7	25.0
ODI (events/h)	7.3 (12.4)	3.6 (3.2)
Obstructive apnea index (events/h)	2.5 (4.6)	1.1 (2.3)
Central apnea index (events/h)	1.1 (2.8)	0.5 (0.7)

AHI: apnea hypopnea index.

ODI: oxygen desaturation index.

No significant differences were found between those with successful versus failed recordings.

**Table 3:** PM data for PD patients and non-PD controls.

	PD patients (*n* = 28)	Controls (*n* = 56)	Adjusted *p* values^*∗*^
Quality variables			
Recording time	470.7 (75.7)	439.9 (84.6)	0.11
Airflow signal quality (%)^*♯*^	91.1 (14.2)	98.3 (5.2)	0.001
Oxygen saturation signal quality (%)^*♯*^	93.4 (16.6)	95.7 (14.6)	0.51
Pulse signal quality (%)^*♯*^	93.9 (16.2)	95.8 (14.6)	0.58
OSA variables			
RDI (events/h)	15.0 (15.1)	22.3 (19.5)	—
Supine RDI (events/h)	18.8 (24.4)	24.2 (21.7)	—
Nonsupine RDI (events/h)	9.6 (12.4)	15.6 (17.6)	—
Time in supine (%)	52.6 (30.8)	48.2 (35.3)	—
Mean saturation (%)	93.7 (4.6)	93.6 (2.5)	—
Oxygen desaturation index (events/h)	6.5 (8.1)	12.6 (13.7)	—

Values are mean (SD) unless specified.

^*♯*^(%) Percentage of optimal signal quality as provided by the RemLogic software.

^*∗*^Adjusted *p* value was obtained by performing linear regression, adjusted for age and gender.

PD: Parkinson's disease.

RDI: respiration disturbance index.

**Table 4:** Correlation of signals quality with age in PD patients and controls.

	Cases		Controls	
	*r*	*p*	*r*	*p*
Airflow signal quality	−0.36	0.06	−0.14	0.32
Oxygen saturation signal quality	−0.36	0.07	−0.11	0.41
Pulse signal quality	−0.34	0.09	−0.10	0.45

*r*: Pearson correlation coefficient.

*p*: *p* value.

**Table 5:** Performance of PM for multiple AHI cut-offs and ODI.

Parameters (*n* = 28)	Sensitivity (95% CI)	Specificity (95% CI)	PPV (95% CI)	NPV (95% CI)	Accuracy (95% CI)
AHI ≥ 5/h (*n* = 25)	**RDI ≥ 5/h**				
	0.84 (0.64–0.95)	0.67 (0.09–0.99)	0.95 (0.77–0.99)	0.33 (0.04–0.78)	0.82 (0.63–0.94)

AHI ≥ 15/h (*n* = 21)	**RDI ≥ 10/h**				
	0.62 (0.38–0.82)	0.71 (0.29–0.96)	0.87 (0.59–0.98)	0.38 (0.14–0.68)	0.64 (0.44–0.81)
	**RDI ≥ 15/h**				
	0.33 (0.14–0.57)	0.71 (0.29–0.96)	0.78 (0.40–0.97)	0.26 (0.09–0.51)	0.43 (0.24–0.63)
	**RDI ≥ 20/h**				
	0.33 (0.15–0.57)	1.00 (0.59–1.00)	1.00 (0.59–1.00)	0.33 (0.15–0.57)	0.50 (0.31–0.69)

AHI ≥ 30/h (*n* = 9)	**RDI ≥ 30/h**				
	0.56 (0.21–0.86)	1.00 (0.82–1.00)	1.00 (0.48–1.00)	0.83 (0.61–0.95)	0.86 (0.67–0.96)

ODI_PSG_ ≥ 5/h (*n* = 5)	**ODI**_**PM**_ ** ≥ 5/h**				
	0.88 (0.47–0.99)	0.80 (0.56–0.94)	0.64 (0.31–0.89)	0.94 (0.71–1.00)	0.82 (0.63–0.94)

RDI (respiration disturbance index) was used for home portable monitoring.

AHI (apnea hypopnea index) was used for polysomnography.

ODI: oxygen desaturation index.

95% CI: 95% confidence interval.

PPV: positive predictive value.

NPV: negative predictive value.

**Table 6:** Assessment of patient characteristics in relation to discrepancies between RDI and AHI.

	RDI/AHI ratio		*p* value	95% CI
Age^*∗*^	<Median (*n* = 14)	>Median (*n* = 14)		
	0.78 (0.84)	0.71 (0.54)	0.82	−0.49; 0.61

MOCA	<26 (*n* = 12)	≥26 (*n* = 16)		
	0.70 (0.82)	0.77 (0.61)	0.81	−0.52; 0.66

Motor UPDRS	<Median (*n* = 14)	>Median (*n* = 14)		
	1.02 (0.85)	0.46 (0.32)	0.03	0.05; 1.08

Dysautonomia^¶^	Yes (*n* = 12)	No (*n* = 16)		
	0.57 (0.31)	0.87 (0.87)	0.22	−0.20; 0.79

Neg. chronotropic med.	Yes (*n* = 6)	No (*n* = 22)		
	0.53 (0.28)	0.79 (0.75)	0.21	−0.17; 0.68

Either dysautonomia or neg. chronotropic med.	Yes (*n* = 18)	No (*n* = 10)		
	0.58 (0.30)	0.89 (0.90)	0.24	−0.22; 0.82

^*∗*^Groups of age were separated according to the median age (63.5 years). Patients with younger age are <63.5 years and patients with older age are ≥63.5 years.

MOCA: Montreal Cognitive Assessment.

UPDRS: Unified Parkinson's Disease Rating Scale.

^¶^The dysautonomia score is based on a question from the first part of the UPDRS (see Section 2), regarding light-headedness on standing. Patients with reported dysautonomia have slight to severe symptoms, whereas patients with no reported dysautonomia have no symptoms of light-headedness.

Neg. chronotropic med.: Negative chronotropic medication (i.e., calcium channel blockers and/or beta-blockers).

RDI: respiratory disturbance index (measured with PM).

AHI: apnea hypopnea index (measured with PSG).

95% CI: 95% confidence intervals.

## References

[1] Barone P., Antonini A., Colosimo C. (2009). The PRIAMO study: a multicenter assessment of nonmotor symptoms and their impact on quality of life in Parkinson's disease. *Movement Disorders*.

[2] Neikrug A. B., Liu L., Avanzino J. A. (2014). Continuous positive airway pressure improves sleep and daytime sleepiness in patients with Parkinson disease and sleep apnea. *Sleep*.

[3] Cochen De Cock V., Abouda M., Leu S. (2010). Is obstructive sleep apnea a problem in Parkinson's disease?. *Sleep Medicine*.

[4] Maria B., Sophia S., Michalis M. (2003). Sleep breathing disorders in patients with idiopathic Parkinson's disease. *Respiratory Medicine*.

[5] Chotinaiwattarakul W., Dayalu P., Chervin R. D., Albin R. L. (2011). Risk of sleep-disordered breathing in Parkinson's disease. *Sleep & Breathing*.

[6] Kendzerska T., Mollayeva T., Gershon A. S., Leung R. S., Hawker G., Tomlinson G. (2014). Untreated obstructive sleep apnea and the risk for serious long-term adverse outcomes: a systematic review. *Sleep Medicine Reviews*.

[7] Mery V. P., Lafontaine A. L., Robinson A. (2013). Impact of sleep disordered breathing on non-motor symptoms of patients with Parkinson's disease. *American Journal of Respiratory and Critical Care Medicine*.

[8] Blackman A., McGregor C., Dales R. (2010). Canadian Sleep Society/Canadian Thoracic Society position paper on the use of portable monitoring for the diagnosis of obstructive sleep apnea/hypopnea in adults. *Canadian Respiratory Journal*.

[9] Collop N. A., Anderson W. M., Boehlecke B. (2007). Clinical guidelines for the use of unattended portable monitors in the diagnosis of obstructive sleep apnea in adult patients. Portable monitoring task force of the American academy of sleep medicine. *Journal of Clinical Sleep Medicine*.

[10] Flemons W. W., Douglas N. J., Kuna S. T., Rodenstein D. O., Wheatley J. (2004). Access to diagnosis and treatment of patients with suspected sleep apnea. *American Journal of Respiratory and Critical Care Medicine*.

[11] Shayeb M. E., Topfer L.-A., Stafinski T., Pawluk L., Menon D. (2014). Diagnostic accuracy of level 3 portable sleep tests versus level 1 polysomnography for sleep-disordered breathing: a systematic review and meta-analysis. *Canadian Medical Association journal*.

[12] Masa J. F., Corral J., Pereira R. (2011). Effectiveness of home respiratory polygraphy for the diagnosis of sleep apnoea and hypopnoea syndrome. *Thorax*.

[13] http://www.instantdiagnostic.com/ids/(S(tkhmzcopwbcxsswjwz2d2rxs))/content.aspx?content=dme_home_sleep_testing_policies.

[14] Smith L. A., Chong D. W. S., Vennelle M., Denvir M. A., Newby D. E., Douglas N. J. (2007). Diagnosis of sleep-disordered breathing in patients with chronic heart failure: evaluation of a portable limited sleep study system. *Journal of Sleep Research*.

[15] Cascon J. A., Pamidi S., Lachapelle P., Kimoff R. J. (2014). Does scoring of autonomic hypopneas improve the diagnostic accuracy of type 3 home sleep recordings in patients with high pre-test probability of obstructive sleep apnea?. *B67. Diagnosis, Treatment, and Management of Sleep Disordered Breathing*.

[16] Hughes A. J., Daniel S. E., Kilford L., Lees A. J. (1992). Accuracy of clinical diagnosis of idiopathic Parkinson's disease: a clinico-pathological study of 100 cases. *Journal of Neurology Neurosurgery and Psychiatry*.

[17] Reichmann H. (2010). Clinical criteria for the diagnosis of Parkinson's disease. *Neuro-Degenerative Diseases*.

[18] Miller M. R., Crapo R., Hankinson J. (2005). General considerations for lung function testing. *The European Respiratory Journal*.

[19] Goetz C. G., Fahn S., Martinez-Martin P. (2007). Movement disorder society-sponsored revision of the unified Parkinson's disease rating scale (MDS-UPDRS): process, format, and clinimetric testing plan. *Movement Disorders*.

[20] Gill D. J., Freshman A., Blender J. A., Ravina B. (2008). The Montreal cognitive assessment as a screening tool for cognitive impairment in Parkinson's disease. *Movement Disorders*.

[21] Iber C., Ancoli-Israel S., Chesson A., Quan S. F. (2007). *The AASM Manual for the Scoring of Sleep and Associated Events: Rules, Terminology and Technical Specifications*.

[22] The Report of an American Academy of Sleep Medicine Task Force (1999). Sleep-related breathing disorders in adults: recommendations for syndrome definition and measurement techniques in clinical research. *Sleep*.

[23] Povitz M., Kimoff R. J. (2014). Use of a level 3 portable monitor for the diagnosis and management of sleep-disordered breathing in an inpatient tertiary care setting. *Canadian Respiratory Journal*.

[24] Ling I. T., James A. L., Hillman D. R. (2012). Interrelationships between body mass, oxygen desaturation, and apnea-hypopnea indices in a sleep clinic population. *Sleep*.

[25] Lachapelle P., Cascon J. A., Pamidi S., Lavigne L., Kimoff R. J. (2015). Autonomic hypopnea scoring and the diagnostic accuracy of home sleep recordings for obstructive sleep apnea: effect of negative chronotropic medication. *D30. For Success Choose the Best: New Tools to Identify Sleep Disordered Breathing*.

[26] Valko P. O., Hauser S., Sommerauer M., Werth E., Baumann C. R. (2014). Observations on sleep-disordered breathing in idiopathic Parkinson's disease. *PLoS ONE*.

[27] Ahmadi N., Shapiro G. K., Chung S. A., Shapiro C. M. (2009). Clinical diagnosis of sleep apnea based on single night of polysomnography vs. two nights of polysomnography. *Sleep & Breathing*.

[28] Trotti L. M., Bliwise D. L. (2010). No increased risk of obstructive sleep apnea in Parkinson's disease. *Movement Disorders*.

[29] Mery V., Lafontaine A., Robinson A. (2013). Treatment of obstructive sleep apnea with continuous positive airway pressure improves non-motor symptoms in Parkinson's disease patients. *Journal of Parkinson's Disease*.

