# Supplementary material for: Diagnosis of Obstructive Sleep Apnea in Parkinson's Disease Patients: Is Unattended Portable Monitoring a Suitable Tool?
Source: Parkinsons Dis. 2015 Oct 13;2015:258418. doi: 10.1155/2015/258418 (PMC4621364; doi:10.1155/2015/258418)
Supplement: Supplementary file 1 — Table 7 in Supplementary Material describes the correlation between the portable monitoring signals quality with several Parkinson's disease associated variables. [file 258418.f1.pdf]

Supplementary materials

Table 7. Correlation of signals quality with PD variables

	H&Y score		PD duration		UPDRS motor		MoCA score		Dysautonomia	
	r.	P-values	r.	P-values	r	P-values	r	P-values	r	P-values
Airflow signal quality	-0.10	0.62	-0.16	0.41	-0.11	0.58	0.17	0.40	0.12	0.54
Oxygen saturation signal quality	-0.24	0.24	0.13	0.52	-0.19	0.37	0.12	0.55	0.14	0.48
Pulse signal quality	-0.22	0.28	0.16	0.44	-0.16	0.44	0.13	0.54	0.19	0.36

PD: Parkinson's Disease

r. : Correlation coefficient

H&Y: Hoehn and Yahr score for PD staging

PD duration: number of years since PD diagnosis

UPDRS motor: motor part of the Unified Parkinson's Disease Rating Scale

MoCA: Montreal Cognitive Assessment
